# Crisis Intervention in a Local Community Emergency Department Inspires Growth of Peer Support Services

**DOI:** 10.5811/westjem.60600

**Published:** 2023-12-20

**Authors:** Phillip Moschella, Mirinda Gormley, Sarah Fabiano, Christopher Carey, Karen Lommel, Jess Hobbs, Rich Jones, Alain H. Litwin

**Affiliations:** * University of South Carolina School of Medicine Greenville, Department of Emergency Medicine, Greenville, South Carolina; † University of South Carolina School of Medicine Greenville, Department of Psychiatry, Greenville, South Carolina; ‡ FAVOR Faces and Voices of Recovery, Greenville, South Carolina; § University of South Carolina School of Medicine Greenville, Department of Internal Medicine, Greenville, South Carolina

In early 2018, the opioid epidemic slammed into the state of South Carolina precipitating an unprecedented rise in overdoses. In response, our local health system partnered with state agencies to implement a novel crisis intervention named the Faces and Voices of Recovery (FAVOR) Overdose Recovery Coaching Evaluation (FORCE). This was the first program in the state to use certified peer support specialists (CPSS) as supplied from a local recovery organization (FAVOR) to engage with overdose patients in the emergency department (ED). Supported by the South Carolina Department of Alcohol and Other Drug Abuse Services, the program ran from January 1–December 31, 2018 within a local community ED in Greenville, South Carolina (∼95,000 adult patients/year).

FAVOR is a community-subsidized, “free” local recovery organization, accredited by the Council on the Accreditation of Peer Recovery Support Services. The CPSSs are individuals who have been in active recovery for at least one year and received training as a CPSS and an assertive community engagement specialist certified by the National Certification Commission for Addiction Professionals and who is a member of the National Association of Alcohol and Drug Abuse Counselors.

All adult patients aged 18 and older, who presented to the ED with an unintentional or accidental opioid overdose were approached to participate in this institutional review board-approved intervention. Patients with an intentional overdose or suicidal intent, known pregnancy, and individuals in police custody were excluded.

Education on the intervention was provided to medical staff through various presentations and email notifications. A “Best Practice Advisory” alert was created in the electronic health record (Epic Systems Corporation, Verona, WI) and would trigger a referral order with additional instructions to call the CPSS when any medical staff member ordered naloxone or documented a diagnosis of opioid use disorder, withdrawal, or accidental overdose. An on-call CPSS would arrive within 30 minutes and, using motivational interviewing, match the patient to an array of resources including the following: 12-step programs; counseling; detox; inpatient rehabilitation; sober living; recovery coaching; and medication-assisted treatment (MAT). The CPSSs collected verbal informed consent, but participants could accept recovery services without consent. A total of 182 patients were approached, and 178 (98%) agreed to participate. Of those patients 109 (61%) were linked with services from the ED, and 114 (64%) remained actively engaged with CPSSs one year later. Fifteen patients (8.4%) returned to the hospital for any reason, and three (1.6%) died of overdose. Reported living situation of participants, at time of index visit, were as follows: 67/178 (38%) with family; 47 (26%) on their own; 25 (14%) homeless; 15 (8%) in a residential recovery center; three (1.70.5%) in “shelters”; one (0.6%) in jail; and 22 (12%) declined to answer.

This data including a 98% initial engagement and 64% remaining actively engaged at one year were compelling. Initially, emergency clinicians and leadership were reluctant to integrate CPSSs into the clinical environment over concerns that patients presenting with an overdose would refuse a consultation and because of a negative perception of the CPSS’s capabilities. By the end of this intervention, team members recognized the importance of CPSSs so much so that during the COVID-19 pandemic, FAVOR CPSSs were offered vaccination within the same priority group as the rest of the ED staff. Overall, this crisis intervention provided crucial experience and intriguing preliminary data that inspired Prisma Health to initiate the following programs through both external grants and internal funding: 1) launching a network of outpatient buprenorphine treatment programs (both clinic-based and mobile units) that have capacity to see uninsured patients (HRSA:HB147075); 2) implementing Screening, Brief Intervention and Referral to Treatment (SBIRT) programs within our busiest urban and rural EDs including seven full-time CPSSs to engage patients and provide harm reduction with take-home naloxone and 3) ED-based MAT initiation (National Institutes of Health HEAL Initiative:NCT05123027, and SAMHSA:H79TI083300). Overall, while CPSSs have spread across the state in multiple EDs and other SBIRT programs their results have been mixed.^1^ This FORCE intervention did supply preliminary data for the CTN multisite “PILOT” trial (CTN-107), which is investigating two models of care using CPSSs across both short-term and long-term CPSS engagement (FORCE model). Prisma Health is one of three participating sites. Overall, this clinical intervention provided the foundation for growth of addiction-related services within our health system and provided preliminary data supporting a National Institute on Drug Abuse-sponsored nationwide clinical trial.
